# Reviewing a Decade of Research Into Suicide and Related Behaviour Using the South London and Maudsley NHS Foundation Trust Clinical Record Interactive Search (CRIS) System

**DOI:** 10.3389/fpsyt.2020.553463

**Published:** 2020-11-27

**Authors:** André Bittar, Sumithra Velupillai, Johnny Downs, Rosemary Sedgwick, Rina Dutta

**Affiliations:** ^1^Institute of Psychiatry, Psychology and Neuroscience, King's College London, London, United Kingdom; ^2^South London and Maudsley NHS Foundation Trust, London, United Kingdom

**Keywords:** electronic health records, natural language processing, machine learning, suicide attempted, suicide completed, self-injurious behaviour

## Abstract

Suicide is a serious public health issue worldwide, yet current clinical methods for assessing a person's risk of taking their own life remain unreliable and new methods for assessing suicide risk are being explored. The widespread adoption of electronic health records (EHRs) has opened up new possibilities for epidemiological studies of suicide and related behaviour amongst those receiving healthcare. These types of records capture valuable information entered by healthcare practitioners at the point of care. However, much recent work has relied heavily on the structured data of EHRs, whilst much of the important information about a patient's care pathway is recorded in the unstructured text of clinical notes. Accessing and structuring text data for use in clinical research, and particularly for suicide and self-harm research, is a significant challenge that is increasingly being addressed using methods from the fields of natural language processing (NLP) and machine learning (ML). In this review, we provide an overview of the range of suicide-related studies that have been carried out using the Clinical Records Interactive Search (CRIS): a database for epidemiological and clinical research that contains de-identified EHRs from the South London and Maudsley NHS Foundation Trust. We highlight the variety of clinical research questions, cohorts and techniques that have been explored for suicide and related behaviour research using CRIS, including the development of NLP and ML approaches. We demonstrate how EHR data provides comprehensive material to study prevalence of suicide and self-harm in clinical populations. Structured data alone is insufficient and NLP methods are needed to more accurately identify relevant information from EHR data. We also show how the text in clinical notes provide signals for ML approaches to suicide risk assessment. We envision increased progress in the decades to come, particularly in externally validating findings across multiple sites and countries, both in terms of clinical evidence and in terms of NLP and machine learning method transferability.

## Introduction

### Suicidality Research Prior to CRIS

Prior to the introduction of electronic health records (EHRs), the study of suicidality in Camberwell, the southeast London catchment area served by King's College Hospital, was undertaken by paper case note review, for example of all referrals to a self-harm team over a 6 month period (1). Data was painstakingly extracted and checked from each consecutive referral to ensure they fitted written criteria and in the Neeleman et al. (1) study a single research question about ethnic differences was posed.

Later, when Dutta et al. (2–4) were trying to determine the epidemiology of completed suicides in a clinically representative cohort of patients experiencing their first episode of psychosis over a 40-year inception period, it was imperative that diagnostic consistency was stringent. They achieved this by amalgamating the Camberwell Cumulative Psychiatric Case Register for the period between January 1, 1965, and December 31, 1983 (5), and then for the period between January 1, 1984, and December 31, 2004, using the basic hospital computer records held at the time with structured fields, to generate a list of all patients admitted with any possible psychotic illness (according to ICD-9 and ICD-10 codes). They then used the information gleaned from reading through the paper case records of all these patients, including medical, nursing, social work, and occupational therapy notes, together with all correspondence relating to the year after each patient's first presentation to complete the Operational Checklist for Psychotic Disorders (OPCRIT) (6). This is a well-validated symptom checklist which enabled operational research diagnostic criteria (RDC) (7) computer diagnoses to be made using the OPRCIT program.

This methodology meant inclusion in the cohort was clearly and consistently defined, and the outcome of deaths by suicide and open verdicts up until March 31, 2007 according to the International Classification of Diseases (ICD) was identified by a direct case-tracing procedure with the Office for National Statistics (ONS) for England and Wales and the General Register Office (GRO) for Scotland. This enabled the study of early risk factors for suicide in the cohort (3) and also studies of both unnatural and natural causes of mortality in first episode psychosis patients (4).

OPCRIT+ (a redesigned version of OPCRIT for use in clinical settings with an expanded number of objectively rated items) facilitated access to structured symptom information entered by clinicians to generate diagnoses including “suicidal ideation” but not self-harm (8), limiting its application for the study of self-harm and suicidal behaviour. However, another more cogent reason for it not being as useful as hoped was “clinicians may feel that these documents are overly prescriptive and restrict their clinical freedom.” There was “disgruntlement amongst the clinicians using the form; extra time on ‘paperwork' is rarely popular” (9) and the OPCRIT+ remains a research tool to obtain “gold standard” research diagnoses, e.g., (10).

### Why EHRs and CRIS?

The widespread adoption of EHRs has meant that large-scale clinical data are now available for clinical research, although researchers have to contend with the large volume, complexity and heterogeneity of these “big data” resources. Typical EHR systems store patient data in both structured fields and as unstructured text (as well as other media types, such as medical images). Structured data fields, such as drop-down menus, forms and checkboxes, tend to be made available to clinical practitioners as a means to directly encode patient diagnoses, assessment results, etc. in a predetermined format. However, rates of completion can vary. Unstructured text entry allows for more nuanced documentation, providing context to assessments, patient status, and other information pertinent to the clinical interaction. The availability of these electronic health data has greatly facilitated mental health research. Investigators can now use EHRs to gather data about clinical populations, identify participants for clinical trials, carry out retrospective case-control studies, develop and trial predictive models, and guide the implementation of evidence-based practices (11, 12).

In 2008, the South London and Maudsley National Health Service (NHS) Foundation Trust Biomedical Research Center (SLaM BRC) developed the Clinical Record Interactive Search (CRIS) application. Since 2008, CRIS became an extensive UK-based repository of anonymised, structured and free-text data derived from the EHR system used by SLaM [See (13) for further details]. Under a strict governance model, CRIS has provided secure access to the de-identified records of all those patients in contact with SLaM services. SLaM provides comprehensive mental health services to an ethnically and socioeconomically diverse population of over 1.2 million residents of all ages, covering four inner city and suburban London boroughs — Croydon, Lambeth, Lewisham and Southwark. SLaM also provides highly specialist services which treat patients from across the UK. SLaM CRIS has been the UK exemplar for all NHS Mental Health Trusts, providing an approach for transforming the electronic health record into a data asset and research tool. The SLaM based CRIS system has been replicated in 12 NHS Trusts across the UK^1^ capturing over 2.6 million patients.

CRIS provides unprecedented information on mental disorders and outcomes in routine clinical care at scale, particularly through enhancements from the use of natural language processing (NLP) to extract previously inaccessible information, ranging from patients' cognitive function, smoking status and education, to antipsychotic medication profiles and substance misuse (14), as well as linkages to external data sources such as national mortality data from the ONS (15), education data (National Pupil Database) (16), and Hospital Episode Statistics (HES) (17). CRIS has also allowed smaller-scale linkages, such as SHIELD, a service improvement project investigating self-harm at the emergency departments of two major London hospitals (18).

The availability of this type of large-scale data heralds the prospect of using statistical and data science approaches to analyse larger cohorts and better understand how these behaviours manifest in healthcare settings (19). However, using these data also presents major challenges, as much of the key clinical information, including suicidal behaviour, is recorded as unstructured clinical case notes and correspondence (20–22).

Over the last 10 years, researchers have used CRIS to conduct a number of epidemiological studies to examine suicidal behaviours across a range of mental health conditions (e.g., autism, psychotic disorders), and demographic groups (e.g., adults, children and adolescents, pregnant women). Methodologies have evolved, improving the accuracy of identifying suicidality-related constructs and predictive models of suicide risk. In the following sections, we review the evidence generated from CRIS on suicidal behaviours, the NLP methods used, and the value of the resulting cohorts and datasets created.

## Identification and Prevalence Estimates of Suicidality in CRIS Clinical Populations

Suicide-related behaviour is the manifestation of a complex set of phenomena that depend on many contextual factors which can change quickly from 1 day to another. Completed suicide remains relatively rare, meaning that tools to assess suicide risk must have a high predictive validity to be of use in a clinical setting (23). Accurate identification of suicide-related behaviour is, therefore, both highly challenging and of prime importance in determining prevalence of suicidal behaviour in clinical populations, and for the development of risk models. While the earliest studies on suicide and related behaviour in CRIS relied on structured fields and mortality data linkages to identify cohorts, increasing efforts have focussed on using NLP to identify suicidality-related concepts in the high volume of unstructured clinical text held in the database. The task of automatically identifying mentions of suicidal behaviour in clinical notes is complicated by the necessity to distinguish actual events relating to the patient from negated mentions, behaviour reported as family history, or those that are recorded with a degree of uncertainty (24). Furthermore, given the inherent variation across clinical populations, which is reflected in the language used in clinical reporting, NLP tools developed for one clinical subpopulation, such as working age adults, may not be reliably transferable to another group, such as school age children, without adaptation. NLP systems used to identify suicide-related constructs in clinical notes must, therefore, be developed for and validated within each target population.

A wide range of known risk and contributory factors are associated with suicide, with symptoms of mental illness being recognisable in more than 85% of people who die by suicide, according to psychological autopsy interviews with family, friends and medical professionals (25, 26). Over the last 10 years, research using CRIS has been conducted to examine the associations of self-harm, suicidality and death by suicide with mental health conditions and a broad range of situational factors, from homelessness to drug misuse to limited service continuity (27–29). As we describe in our summary below, initial studies on suicide and related behaviour in CRIS used structured fields held within standard assessment forms or diagnostic codes. Progressively, researchers began to make use of CRIS's free-text fields and search functionalities, while more recently, NLP techniques have been employed to extract and structure suicide-related information from within the case notes. The principal characteristics of the clinical cohorts mentioned in this review are summarised in Table 1.

**Table 1:** Summarised characteristics of clinical cohorts created using CRIS for the study of suicide and related behaviour.

**Study**	**Clinical group**	**Population size**	**Number of events**	**Age**	**Date range**	**Method to identify suicide and related behaviour**
Polling et al. (18)	Adults attending ED	7,444	10,688 ED attendances	N/A	01/04/2009–31/12/2011	ICD-10 codes X60-X84, presence of keywords related to self-harm, suicide attempts and suicidality
Bogdanowicz et al. (28)	Patients with opioid use disorder	5,335	N/A	15–73 years Mean (SD) = 37.6 (9.07) years	01/04/2008–31/03/2014	ICD-10 codes X409-X450, Y120, Y125, F119^†^
Lopez-Morinigo et al. (30)	Patients with schizophrenia spectrum disorder	426 (71 cases, 355 controls)	N/A	Mean (SD) = 44.9 (18.0) years	01/01/2007–31/12/2013	ICD-10 codes X64, X70, X71, X78, X80, X81, X84, Y10-34
Lopez-Morinigo et al. (31)	Patients accessing secondary mental healthcare	13,758	N/A	Mean (SD) = 41.3 (12.2) for suicide, 40.6 (11.5) for no suicide	01/01/2007–01/04/2015	ICD-10 codes X64, X70, X71, X78, X80, X81, X84, Y10-34
Roberts et al. (32)	Individuals with chronic fatigue syndrome	2,147	N/A	Mean = 39.1 years	01/01/2007–31/12/2013	ICD-10 codes X60-X84
Taylor et al. (33)	Perinatal women with SMI	420	N/A	Mean (SD) = 31.9 (6.2) years	01/01/2007–31/12/2011	Presence of keywords [from (18)] related to self-harm, suicide attempts and suicidality
Downs et al. (34)	Children and adolescents with ASD	1,906	N/A	14–18 years	01/01/2008–31/12/2013	NLP, manual classification of suicidality-related expressions
Velupillai et al. (35)	Adolescents attending CAMHS	23,455	N/A	11–17 years	01/04/2009–31/03/2016	Manual annotation of suicidality-related expressions, NLP
Bittar et al. (36)	Patients accessing secondary mental healthcare	17,640 (2,913 cases, 14,727 controls)	21,175 admissions (4,235, cases, 16,940 controls)	Mean (SD) = 33.7 (15.6) years	02/04/2006–31/03/2017	X6^*^, X7^*^, X80-4^*^, Y1^*^, Y2^*^, Y30-4^*^, Y87^*^

† *Due to indeterminacy of intent, suicide by overdose and fatal drug poisonings are grouped together*.

* *indicates all codes that begin with the given sequence*.

### Using Structured Data

#### Suicidality Outcome Data

The Health of the Nation Outcome Scales (HoNOS) were introduced in 1996, to measure the health and social functioning of people with mental illness. Within SLaM, as with most UK mental health trusts, clinicians are expected to complete HoNOS for all patients receiving care. The non-accidental self-injury item on the HoNOS score has been shown to be the only individual item associated with higher mental health service costs (37). It has been used in a number of studies in CRIS to assess both the direct and indirect impact of self-harm. The individual non-accidental self-injury HoNOS item has been included as a covariate in a number of analyses of adverse outcomes within CRIS. These include homelessness and length of hospital stay for psychiatric inpatients (27), functional status and mortality in serious mental illness (38), facilitated discharge and bed days (39), and the effects of clozapine on premature mortality (15). When assessing self-harm as a potential risk factor for mortality among patients with personality disorder, the HoNOS item was again used in isolation as a marker of self-harm risk (40). Despite the provision of optional structured questionnaires on CRIS, such as the Patient Health Questionnaire-9 (PHQ-9) (whose final item enquires about thoughts of self-harm and suicide) and the Beck Scale for Suicide Ideation (BSS), very few are completed in general clinical work where free-text input is favoured by clinicians, making them of limited value for studies of real-world clinical cohorts. Conversely local NHS Trust requirements to complete structured suicide risk assessments for all patients means this data is better recorded and has been studied.

#### Suicide Risk Assessment Data

Structured suicide and violence risk assessments in mental health services has been shown to have low predictive accuracy for all-cause mortality (30), however these assessments have continued to be used in clinical practice. Lopez-Morinigo et al. examined the use of risk assessment proforma for their investigation into suicide completion in secondary mental health care. The risk proforma, which clinicians were expected to use at that time according to local clinical policy, consisted of present/absent tick boxes for factors including suicidal history, suicidal ideation and alcohol misuse. They found that patients with a diagnosis other than schizophrenia spectrum disorder who had died by suicide, were much less likely than patients with schizophrenia to either have had a full risk assessment or a complete HoNOS even though they showed increased frequency and greater predictability in key suicide risk assessment factors: suicidal ideation, hopelessness, impulsivity and significant loss (29). In their later study, they found structured risk assessment relating to suicide in schizophrenia spectrum disorders to be of little use in predicting completed suicide, with risk assessments fully completed in only 43.6% of patients who had died by suicide (30). Subsequent work revealed a limited role for structured risk assessment, especially in its usefulness in revealing more nuanced factors relevant to suicide risk such as “mental pain” (31). They suggest that research should “switch the focus from long-term risk factors to short-term risk algorithms, which are more relevant to the clinician.”

#### Suicide Mortality Data

Research into mortality, including death by suicide, has typically utilised ICD-10 diagnostic codes (which must be completed as part of clinical assessment), linked with outcome data from the Office for National Statistics, ONS (15, 41). In a retrospective cohort study, Roberts et al. (32) used CRIS to investigate the mortality of individuals in secondary and tertiary care who had been diagnosed with chronic fatigue syndrome (CFS). Although all-cause mortality for people with CFS was not significantly different to that of the general population, there was a significantly elevated risk of completed suicide. CRIS has also been used to conduct a number of pharmaco-epidemiological studies, for example [Hayes et al. (15)] examined the risk or potential risk mitigation of psychopharmacological interventions on death by suicide in patients with serious mental illness (including schizophrenia, schizoaffective and bipolar disorders). Findings of this study demonstrated treatment with the medication clozapine was associated with a reduction in risk of death by unnatural causes, including suicide, as well as natural causes.

### Using Unstructured Data

#### Free-Text Keywords to Study Self-Harm Presentations to Emergency Departments

Polling et al. (18) used external data linkages in combination with CRIS data (including keywords recorded in free-text fields) to create a novel dataset for the study of self-harm, which is strongly associated with mental health disorders, and is the strongest single risk factor for future suicide. In England, population-level assessment of self-harm is recorded in the Hospital Episode Statistics (HES) database. However, many emergency department attendances, namely those that do not lead to a hospital admission, still go unrecorded in HES, and completion of the reason for presentation is low, thus limiting the value of this data source for studies of self-harm presentations. Polling et al. addressed these shortcomings by combining routinely collected data from electronic health records in CRIS and HES. They validated their data against another dataset curated through manual review of emergency department notes and audit forms, also compiling a list of self-harm search terms.

#### Free-Text Keywords to Study Perinatal Self-Harm in Women With Psychiatric Disorders

Using the self-harm-related terms identified by Polling et al. (18) and Taylor et al. (33) investigated the prevalence and risk factors of self-harm and suicide ideation in women with psychotic disorders and bipolar disorder during pregnancy. They identified a cohort of 420 patients by performing a free-text search of CRIS records for both suicidal ideation and self-harm. The perinatal period is generally associated with lower risk of both suicide and self-harm in the general population, however, women diagnosed with severe postpartum psychiatric disorders are up to 70 times more at risk of suicide. In Taylor et al.'s cohort, 24.3% of women had a report of suicidal ideation and 7.9% had a recorded self-harm event during their index pregnancy.

#### Free-Text Keywords to Study Self-Harm and Human Trafficking

In a further study using the free-text search capabilities of CRIS, Borschmann et al. (42) carried out an analysis of self-harm among victims of human trafficking. They identified patients for their cohort by searching the CRIS free-text notes for terms indicating possible trafficking (e.g., “victim of trafficking,” “sex trafficking,” “trafficked”). In the same way, documents were screened for mentions of self-harm behaviour using a list of terms including “self-harm,” “DSH,” “burn^*^” and “electrocut^*^.” They found that 33% of all trafficked patients had engaged in self-harm prior to care, while 25% did so during care. After self-harming, trafficked patients were subsequently more likely to be admitted to a ward than those who had not been victims of human trafficking. After self-harming, trafficked patients were more likely than non-trafficked patients to be admitted as a psychiatric inpatient, but less likely to attend an emergency department.

### Using Natural Language Processing (NLP)

The first approaches that were developed to process CRIS data were pattern matching approaches to identify certain pieces of information (e.g., medication, smoking status, substance misuse) using the GATE framework (43). In many cases, the information of interest is a particular clinical construct (e.g., hallucinations, echolalia) or a specific diagnosis. A bespoke application, called TextHunter (14), was developed for these types of constructs. TextHunter is a software application that requires a set of manually pre-annotated examples to train a supervised machine learning classifier (Support Vector Machine). These NLP applications identify and classify the relevant constructs and produce structured variables indicating their presence or absence within the texts. These structured variables are stored in table columns in the CRIS database. Researchers may access these variables (along with the “standard” structured fields – e.g., diagnosis codes, demographic information, dates – from the EHR) through the SQL interface of the CRIS database to identify cohorts of patients for epidemiological studies and clinical research. Several studies cited herein have made use of these structured variables (28, 32, 36).

In addition to these “integrated” NLP applications, clinicians have worked alongside NLP researchers to develop custom NLP tools to identify suicide-related constructs in specific population samples within CRIS. As we have seen, the focus of most work has been the epidemiology and prevalence of suicidal behaviour, with NLP tools that use both rule-based (35, 44) and machine learning paradigms (45), including neural network architectures (46). Most recently, efforts have also been made to model dynamic suicide risk using supervised machine learning (36).

#### Study of Mortality in Opioid Use Disorder Patients Using NLP to Identify Cohorts

Using data from CRIS with an external linkage to ONS mortality data, Bogdanowicz et al. (28) investigated the effectiveness of addiction-specific clinical risk assessments for identifying groups with high mortality in opioid use disorder (OUD). Patients with a diagnosis of OUD were identified by ICD-10 code F11. ICD-10 diagnosis was supplemented with structured output of one of the CRIS NLP tools that identifies diagnoses in unstructured clinical notes. Overdose (both accidental and intentional) was the most common cause of death and clinically assessed suicidality was found to be significantly associated with increased overdose mortality.

#### NLP to Identify Suicide-Related Behaviour

Today, with the increasing body of research on suicide and related behaviour in CRIS, and a diversity of clinical population groups under study, has come a need to develop more targeted methods of accessing the suicide-related data within the unstructured clinical narratives. NLP systems designed for this task need to identify the different types of suicide-related behaviour (suicide attempt, suicidal ideation, self-harm, etc.) and account for the linguistic variation that indicates whether a mention is attested, negated or uncertain, is relevant to the patient, or a family member, and so on. These considerations have spurred on the recent development of bespoke NLP tools. For example, Gkotsis et al. (44) developed an NLP system specifically designed to detect whether a suicide-related concept is negated or not. This system was developed and evaluated on a random sample of clinical notes from CRIS. In a more recent study, Fernandes et al. (45) developed two NLP approaches to detect relevant mentions of suicidal ideation and another to identify recorded suicide attempts.

#### NLP Features to Identify Key Suicide Risk Periods

Identifying periods during which a patient is at elevated risk of making a suicide attempt is key to enabling timely intervention. However, information available to clinicians concerning the rapidly changing dynamic factors leading up to a suicide attempt has been limited. Bittar et al. (36) explored whether it is possible to use EHRs to automatically predict suicide attempts in a broad clinical population (across all age groups) using only data from a relatively short period of 30 days leading up to an event. This work was based on the hypothesis that periods prior to a suicide attempt are a time of acute crisis that is reflected, explicitly or implicitly, in clinician records, making these periods distinguishable from periods not preceding an attempted suicide. Combining all three features of (1) structured data from EHRs, (2) structured values extracted by NLP software, and (3) vectorised bag-of-words of all documents provided the best model to classify or distinguish between “document windows” prior to a suicide attempt or not. Thus, the features were found to be complementary in this study.

#### NLP to Study Suicidal Behaviour in Children and Adolescents

The risk and conceptualisation of suicidal behaviour for children and adolescents can be different to adults (47). Downs et al. (34) conclude that the clinician notes on suicidal risk in children and adolescents are different to an adult review. For example, clinicians may have a greater reliance on third person report, where caregivers voice concerns regarding the young person's suicidality. It is also possible that suicidality is “discovered” rather than being the presenting complaint, hence changing the emphasis and position of suicide-related text/progress notes within the young person's clinical record.

Adolescence is associated with a high risk of suicide and self-harm compared to most other age groups, but few studies have examined the prevalence of suicidal behaviour in large adolescent patient cohorts. Downs et al. (34) first used CRIS to explore suicidality in young people but focussed on a population with autism spectrum disorders (ASD), who have shown much greater risk of suicidal behaviours than neurotypically developing children. A cohort of young people diagnosed with ASD were identified and NLP techniques were used to identify suicidal behaviour from the clinical notes in CRIS. Their corresponding free-text notes (progress reports, medical correspondence, risk assessments, etc.) were manually annotated for mentions of suicidality by clinical researchers. A prevalence analysis of suicidality in a sample of the data showed that only 3% of all documents mentioned suicide-related information.

Using a subset of this cohort, Holden et al. (48) used a historical cohort design and applied NLP approaches to extract information on victimisation by bullying and suicidal behaviour. They found those young people with ASD who were bullied were nearly twice as likely to report later suicidal ideation. The dataset created by Downs et al. has also recently proven useful to train machine learning models for use in suicide research. Song et al. (46) used a revised version of the data to develop a deep neural network classifier that identifies sentences containing positive mentions of suicidality while taking into account the contextual information in surrounding sentences. This type of approach provides an alternative to modelling suicide-related information from text that better takes into account the narrative discourse in the clinical documentation.

Velupillai et al. (19) developed and validated a method for identifying suicidality across a more heterogenous clinical adolescent population in EHRs using NLP, expanding the population beyond ASD. They examined 1,601,422 documents from 23,455 young people and developed a method to accurately identify suicidal behaviour information in a very broad clinical population. The resulting dataset and NLP approaches used, provide a powerful example of how NLP approaches can be used to rapidly examine the prevalence of suicidal behaviour in very large adolescent clinical populations.

#### NLP to Study Depression and Suicidality in Older Adults

Free-text mentions of depressive symptoms were used as outcome measures in the assessment of later-life depression in people from ethnic minorities by Mansour et al. (49). This study used NLP tools designed to detect depressive symptoms recorded in unstructured texts in CRIS, including the identification of mentions of suicidal ideation. These depressive symptom NLP tools, developed to account for the presence of contextual markers such as negation and irrelevant concepts, were also used by Cai et al. (50) in their investigation into predictors of mortality in people with late life depression.

## The Next Ten Years?

Although EHR data are not created for research purposes, they provide a rich resource for large-scale retrospective research, allowing identification of diverse and comprehensive clinical study samples. One of the main challenges in suicide research is obtaining sufficiently large study samples to study an outcome with a high enough base rate for predictive modelling to have a meaningful positive predictive value. The low base rate of completed suicide limits the predictive value of any model, whether established statistical techniques or machine learning (51), but related behaviours, such as suicidal ideation, intention, planning and self-harm can be studied. Over the past 10 years, CRIS has provided an unprecedented resource for studying suicide and related behaviour in a UK clinical population to an extent that would not have been possible before the introduction of EHRs. The development and implementation of this type of resource is an incredibly valuable investment, which should be encouraged.

One avenue of research being pursued in CRIS is comparison of suicide-related phenomenon over a span of time, within the same hospital trust culture, but where mandatory changes have occurred with regards to how assessments are made and recorded. The focus on a single mental health trust for a review opens the opportunity for a different set of more detailed analyses than a review that covers multiple sites (52).

Furthermore, EHRs reflect real-world clinical practice. This means that the context of how, for example, structured risk assessment tools and other schedules, like HoNOS, are used in daily clinical work needs to be well understood when including them as variables in clinical research studies. Most of the relevant information is found in the free text, and appropriate NLP solutions are key components for enabling risk modelling.

Looking to the future, replication studies of work based on SLaM CRIS, including the developed NLP applications, across other EHR systems and in other clinical catchment areas would provide insights into the generalisability of these particular models to new clinical settings. However, the portability of these NLP applications needs further scrutiny. The studies in this review all have developed their methodologies from the same CRIS system; clinical text may have higher internal homogeneity (e.g., in terminology) with respect to other CRIS systems based in other health districts. Testing the generalisability of the NLP tools described in this review across other health organisations is essential and has only just begun. As described earlier, CRIS has also been implemented in other sites across the UK. On example is the Camden & Islington Research Database (53), which has proved a useful starting point for comparison with SLaM CRIS as the data reflects a similar healthcare organisation with socio-demographic and geographic similarities, i.e., represents a comparable, but not identical, urban population.

Other studies using EHR data for suicide-related research range from those that use rule-based approaches to e.g., estimate the use of diagnostic codes vs. information recorded in free text broadly in EHR data (21) or to monitor suicidal patients in primary care (20), to studies using more advanced ML and NLP approaches for e.g., psychiatric readmission risk prediction using inpatient psychiatric discharge summaries where suicidality could be an important risk factor (54), for automated epidemiological surveillance of suicide attempts in emergency departments in France (22), or for estimating risk of death by suicide after discharge (55). Findings from these studies are, however, currently difficult to compare, as the underlying populations, healthcare settings, EHR systems, and data-driven approaches differ.

Furthermore, given the inherent variation across clinical populations, which is reflected in the language used in clinical reporting, NLP tools developed for one clinical subpopulation, such as working age adults, may not be reliably transferable to another group, such as school age children, without adaptation. NLP systems used to identify suicide-related constructs in clinical notes must, therefore, be developed for and validated within each target population. The same principle applies for the application of NLP tools across institutions and EHR systems. The studies in this review all use data from the same system, CRIS, for which language is likely to show a certain level of internal homogeneity (e.g., in terminology) with respect to other systems. Testing the generalisability of the NLP tools described below in this review has only just started.

When also including free text and NLP models, as mentioned above, the extent to which internal homogeneity (e.g., in terminology) impacts results across different institutions and clinical settings, is an area well worth further studies to further advance this field and provide evidence about the broader generalisability of findings. The culture, incentives, and structure of clinical systems outside of the UK may induce further differences between the signals of NLP systems for detecting discussion of suicide. Collaborative efforts are currently being made to compare methodologies and NLP tools across healthcare institutions not just within the UK, but also with collaborators in the USA. We envision advances in ML and NLP methods, standards for interoperability, and infrastructures to enable such comparisons in the future.

Furthermore, advances in computational analysis of EHR data, e.g., machine learning in combination with NLP, will continue to develop, and provide novel solutions to suicide research (56). With the existing CRIS subsets, clinical cohorts, and NLP approaches developed for the studies described in this review, benchmarks have been created that allow for appropriate comparisons between different methodologies.

Going beyond identification or prediction of those at risk, analysis of continuously collected data, and integration of EHR data with smartphone, wearable device and even social media data could allow collection of data across different time periods, not just at the time of clinical interactions, thus helping to understand suicidal crises and enabling delivery of targeted suicide prevention interventions (57).

## Summary and Conclusion

In this review of a decade of research into suicide and related behaviour using CRIS we have summarised the evolution of different methods employed to identify suicide and related behaviour, including linkages to mortality data, structured ICD-10 codes, manual review of clinical notes, keyword searching in free text and relevant mentions identified using NLP techniques. Cohorts under study have varied in size from several hundred to tens of thousands of patients and have covered adult, elderly as well as child and adolescent patients. A range of clinical disorders have been described from the perspective of suicide and related behaviours, including pregnancy, severe mental illness and self-harm, opioid use disorder patients, chronic fatigue syndrome and autism spectrum disorders. Finally, some studies have identified and investigated specific clinical events, such as emergency department attendances or hospital admissions.

In conclusion, the breadth and depth of the research and findings of understanding suicide and related behaviour from this past decade using CRIS have accelerated the field in ways unthinkable prior to the availability of EHR data. These studies not only add to the clinical evidence base, but also reflect an important evolution of data-driven method applicability and development that is central to advancing this field further. We envision increased progress in the decades to come, particularly in externally validating findings across multiple sites and countries, both in terms of clinical evidence and in terms of NLP and machine learning method transferability.

## Gaining Access to CRIS

The de-identified CRIS database has received ethical approval for secondary analysis: Oxford REC C, reference 18/SC/0372. The data is used in an anonymised and data-secure format under strict governance procedures. CRIS data is made available to researchers with appropriate credentials (provided by the South London and Maudsley NHS Trust) working on approved projects. Projects are approved by a CRIS Oversight Committee, a body set up by and reporting to the South London and Maudsley Caldicott Guardian. On request, and after appropriate credentials have been obtained as well as arrangements with the lead of the respective CRIS project, data presented in this study can be viewed within the secure system firewall.

## Author Contributions

RD and SV proposed the manuscript and its contents. AB wrote the first draft of the manuscript and compiled data pertaining to study design, cohorts and NLP systems used in the cited literature, and incorporated edits by other authors. Each author contributed to specific sections of the manuscript: RD and SV on introductory and historical overviews: RS on use of structured data fields: JD on child and adolescent populations: AB and SV on natural language processing: RD, SV, and JD on perspectives and conclusions. All authors contributed to editing and revising the manuscript and approved the final version.

## Conflict of Interest

RD and SV declare previous research funding received from Janssen. The remaining authors declare that the research was conducted in the absence of any commercial or financial relationships that could be construed as a potential conflict of interest.

## Footnotes

^1^See https://crisnetwork.co/uk-cris-programme
